# Kynurenine, Tetrahydrobiopterin, and Cytokine Inflammatory Biomarkers in Individuals Affected by Diabetic Neuropathic Pain

**DOI:** 10.3389/fnins.2020.00890

**Published:** 2020-08-21

**Authors:** Ananda Staats Pires, Benjamin Heng, Vanessa X. Tan, Alexandra Latini, Marc A. Russo, Danielle M. Santarelli, Dominic Bailey, Katie Wynne, Jayden A. O’Brien, Gilles J. Guillemin, Paul J. Austin

**Affiliations:** ^1^Neuroinflammation Group, Department of Biomedical Sciences, Faculty of Medicine and Health Sciences, Macquarie University, Sydney, NSW, Australia; ^2^Laboratório de Bioenergética e Estresse Oxidativo, Departamento de Bioquímica, CCB, Universidade Federal de Santa Catarina, Florianópolis, Brazil; ^3^Hunter Pain Clinic, Broadmeadow, NSW, Australia; ^4^Genesis Research Services, Broadmeadow, NSW, Australia; ^5^Department of Diabetes and Endocrinology, John Hunter Hospital, Newcastle, NSW, Australia; ^6^School of Medicine and Public Health, University of Newcastle, Callaghan, NSW, Australia; ^7^Discipline of Anatomy and Histology, School of Medical Sciences, Faculty of Medicine and Health, The University of Sydney, Sydney, NSW, Australia

**Keywords:** neuropathic pain, kynurenine, type 1 diabetes, tetrahydrobiopterin, pro-inflammatory cytokines

## Abstract

Neuropathic pain is a common complication of diabetes with high morbidity and poor treatment outcomes. Accumulating evidence suggests the immune system is involved in the development of diabetic neuropathy, whilst neuro-immune interactions involving the kynurenine (KYN) and tetrahydrobiopterin (BH4) pathways have been linked to neuropathic pain pre-clinically and in several chronic pain conditions. Here, using a multiplex assay, we quantified serum levels of 14 cytokines in 21 participants with type 1 diabetes mellitus, 13 of which were classified as having neuropathic pain. In addition, using high performance liquid chromatography and gas chromatography-mass spectrometry, all major KYN and BH4 pathway metabolites were quantified in serum from the same cohort. Our results show increases in GM-CSF and IL-8, suggesting immune cell involvement. We demonstrated increases in two inflammatory biomarkers: neopterin and the KYN/TRP ratio, a marker of indoleamine 2,3-dioxygenase activity. Moreover, the KYN/TRP ratio positively correlated with pain intensity. Total kynurenine aminotransferase activity was also higher in the diabetic neuropathic pain group, indicating there may be increased production of the KYN metabolite, xanthurenic acid. Overall, this study supports the idea that inflammatory activation of the KYN and BH4 pathways occurs due to elevated inflammatory cytokines, which might be involved in the pathogenesis of neuropathic pain in type 1 diabetes mellitus. Further studies should be carried out to investigate the role of KYN and BH4 pathways, which could strengthen the case for therapeutically targeting them in neuropathic pain conditions.

## Introduction

Peripheral diabetic neuropathy is a disorder of the peripheral nervous system that preferentially targets sensory axons, autonomic axons and later, to a lesser extent, motor axons (for a review see Feldman et al., 2019). Diabetic neuropathy is responsible for the greatest morbidity in terms of depression, anxiety, loss of sleep and non-compliance with treatment in diabetic patients (Holzer, 1998; Jensen et al., 2007). A sizable proportion of patients with peripheral diabetic neuropathy (25–60%) also develop neuropathic pain (diabetic neuropathic pain; DNP) (Tavakoli and Malik, 2008; Abbott et al., 2011; van Hecke et al., 2014), which is defined as “pain caused by a lesion or disease affecting the somatosensory system” (IASP, 2017). Management of DNP can be challenging for both the clinician and the patient, with pain being unresponsive, or only partially responsive, to existing pharmacological approaches (Jose et al., 2007). Thus, a pressing need exists to develop a greater understanding of the underlying molecular mechanisms responsible for the characteristic intractable chronic pain associated with diabetic neuropathy in order to develop more effective therapies.

Over the last two decades, advances in the understanding of the mechanisms eliciting chronic neuropathic pain have identified a critical interaction between the immune system and the nervous system (Austin and Moalem-Taylor, 2010). The two systems are tightly integrated, cooperating in local and systemic reflexes that restore homeostasis in response to tissue injury and infection. They further share a broad common language of cytokines, growth factors, and neuropeptides that enables bidirectional communication. However, this reciprocal crosstalk permits amplification of maladaptive feedforward inflammatory loops at multiple levels of the neuraxis, contributing to the development of both sensory and emotional aspects of chronic pain (for detailed reviews see Grace et al., 2014; Talbot et al., 2016; Austin and Fiore, 2019). A number of recent studies have identified elevated immune markers (C-reactive protein, tumor necrosis factor alpha (TNF-α), interleukin (IL) 6 (IL-6), toll-like receptor (TLR) 4, transforming growth factor beta 1 (TGF β 1) and the presence of antinuclear and anti-ganglioside auto-antibodies in diabetic neuropathy (Hussain et al., 2013, 2016; Janahi et al., 2015; Zhu et al., 2015; Ge et al., 2016), however these studies do not distinguish between painless and painful neuropathy.

**FIGURE 1:**
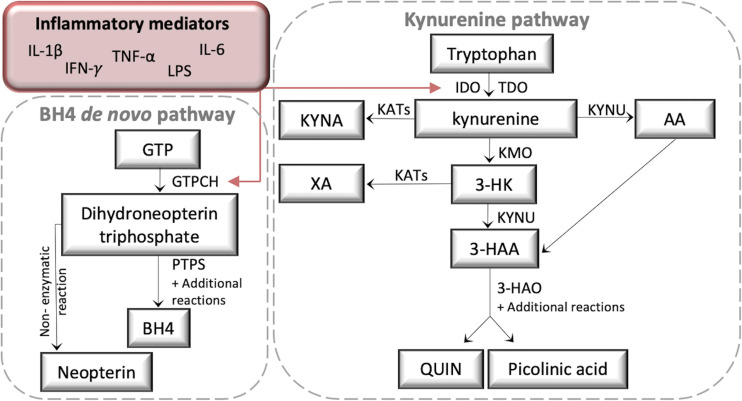
A schematic diagram of the kynurenine (KYN) and tetrahydrobiopterin (BH4) pathways. Both KYN and BH4 pathways are activated by inflammatory cytokines such as IL-1β, Il-6, IFN-γ, TNF-α, and the immune activator LPS. LPS, lipopolysaccharide; GTP, Guanosine-5’-triphosphate; GTPCH, GTP cyclohydrolase I; PTPS, 6-pyruvoyl-tetrahydropterin synthase; IDO, Indoleamine 2,3-dioxygenase; TDO, tryptophan 2,3-dioxygenase; KATs, Kynurenine aminotransferases; KYNA, Kynurenine acid; XA, Xanthurenic acid; KYNU, Kynureninase; AA, Anthranilic acid; KMO, Kynurenine 3-monooxygenase; 3-HK, 3-Hydroxykynurenine; 3-HAA, 3-Hydroxyanthranilic acid; 3-HAO, 3-hydroxyanthranilate 3,4-dioxygenase; QUIN, Quinolinic acid.

The kynurenine (KYN) and tetrahydrobiopterin (BH4) pathways are critical regulators of neuro-immune crosstalk (Staats Pires et al., 2020). Inflammation rapidly activates both pathways, producing several neuroactive metabolites (Figure 1), and there is emerging evidence that KYN pathway (KP) activation contributes to the pathogenesis of chronic pain and co-morbid depression through resultant neuroinflammation and neurotoxicity (Kim et al., 2012; Walker et al., 2014; Zhou et al., 2015; Rojewska et al., 2016, 2018; Laumet et al., 2017). Inflammatory cytokines, such as interferon gamma (IFN-γ) and TNF-α, are activators of indoleamine 2,3-dioxygenase (IDO1), a major biosynthetic enzyme in the conversion of tryptophan (TRP) to KYN. KYN is metabolized by kynurenine 3-monooxygenase (KMO) into 3-hydroxy-kynurenine (3-HK). 3-HK is converted to the neurotoxic metabolite quinolinic acid (QUIN) in both macrophages and microglia by the enzymes kynureninase (KYNU) and 3-hydroxyanthralinic acid dioxygenase (HAO) (Guillemin, 2012). A recent study in over 17000 chronic pain patients identified QUIN as the most commonly elevated biomarker, with the authors suggesting a role in enhanced nociception through peripheral NMDA-receptor activation (Gunn et al., 2020). Moreover, the inhibition of IDO and KMO reduces allodynia and depressive-like behavior in rodent models of neuropathic pain (Rojewska et al., 2016, 2018; Laumet et al., 2017).

GTP cyclohydrolase I (GTPCH), the rate-limiting enzyme of BH4 biosynthesis, is also activated by IFN-γ and TNF-α (Werner et al., 1990, 1993; Connor et al., 2008; Dias et al., 2016). BH4 has recently been identified as a key mediator of chronic pain. Elevated levels have been found in axotomized sensory neurons and macrophages infiltrating injured nerves, whilst inhibiting its production reduces pain sensitivity (Latremoliere et al., 2015; Fujita et al., 2019). BH4 acts as a mandatory cofactor for the production of catecholamines and nitric oxide (for a review see Ghisoni et al., 2015) and it is essential for the effective activation and proliferation of mature T cells (Cronin et al., 2018). Neopterin (NEO) is also downstream of GTPCH, however whilst production of BH4 relies on further enzymatic conversion from 6-pyruvoyl-tetrahydropterin synthase (PTPS) and others, NEO is produced by a non-enzymatic reaction. Consequently NEO is considered a more sensitive biomarker of immune activation than BH4 (Werner et al., 1990, 1993; Ghisoni et al., 2015).

Therefore, measurement of BH4, NEO and KP metabolites in biological fluids may offer relevant information about the progression and pathogenesis of DNP. Here, we present a comprehensive analysis of KP metabolites, as well as BH4 and NEO, in serum from diabetic individuals with chronic neuropathic pain (DNP) compared to diabetic controls with no pain (DC). Since pro-inflammatory cytokines activate KYN and BH4 pathways, we also analyzed a panel of 14 cytokines in the serum of these individuals.

## Materials and Methods

### Participants

Participants were recruited by Genesis Research Services (Broadmeadow, NSW, Australia) between April 2018 and Sept 2019. The study included both male and female participants aged 40–71 years old, with definite clinical diagnosis of type 1 diabetes mellitus (*n* = 21). Thirteen of these participants were in the diabetic neuropathic pain group (DNP), as they had a definitive clinical diagnosis of painful diabetic peripheral neuropathy, whilst the remaining eight participants were classified in the diabetes control group (DC). Classification of the diabetes patients in to the DNP (*n* = 13) or DC (*n* = 8) groups was further confirmed using the Douleur Neuropathique 4 (DN4) questionnaire (Bouhassira et al., 2005), based on the absence (score < 4) or presence (score ≥ 4) of neuropathic pain. Exclusion criteria included age less than 18 years; chronic neuropathic pain lasting less than 3 months; presence of acute pain, non-neuropathic pain, or mixed pain; any neurological, psychiatric or pain condition that could confound the study endpoints; and pregnancy. For the DC group, further exclusion criteria included any evidence of peripheral neuropathy (i.e., numbness, non-painful paresthesias or tingling, non-painful sensory distortions or misinterpretations), or any other microvascular complications of diabetes (i.e., retinopathy, nephropathy). All participants were taking medication to manage their diabetes. On the day of blood collection, participants had ceased taking immune-modulating medications (e.g., NSAIDs, steroids or opioids) for at least 7 days, which was the longest wash-out clinically practicable.

### Ethics Approval and Consent to Participate

This study was approved by the Human Ethics Committee from University of Sydney (HREC #2017/019) and Macquarie University (HREC #5201600401). Authorization for access to participants who fit the inclusion criteria was also granted by the Hunter New England Local Health District. Participation in the study was on a completely voluntary basis, and all participants signed informed consent. All demographic information and blood samples were de-identified from the study team.

### Pain and Psychological Profiling

All participants rated pain intensity from 0 to 100 on a visual analog scale (VAS) and completed 5 questionnaires to assess pain and psychological variables: Short-form McGill Pain Questionnaire (SF-MPQ-2) (Dworkin et al., 2009), Short-form Depression, Anxiety and Stress Scale (DASS21) (Lovibond and Lovibond, 1995), Pain Self-Efficacy Questionnaire (PSEQ) (Nicholas, 2007), Tampa Scale for Kinesiophobia (TSK) (Roelofs et al., 2011) and Pain Catastrophizing Scale (PCS) (Sullivan et al., 1995).

### Blood Collection Protocol

A non-fasted blood sample was taken by venepuncture from the antecubital fossa by a trained phlebotomist into a 5 mL tube. Samples were then incubated at room temperature for 15 min to allow the blot to clot, then centrifuged at 2,000 × *g* for 10 min. The supernatant was removed and stored in polypropylene aliquot tubes at −80°C until analysis.

### Sample Preparation

Serum samples were deproteinised by adding 1 vol. of 10% trichloroacetic acid containing 6.5 mM dithioerythritol. Afterward, samples were centrifuged at 12,000 × *g* at 4^*o*^C for 15 min and filtered through a 0.20 μm PTFE syringe filter (Merck-Millipore, CA, United States). Supernatants were transferred to high performance liquid chromatography (HPLC) vials for analysis.

### Metabolites Quantification by HPLC

Serum levels of xanthurenic acid (XA), TRP, KYN, 3-HK, 3-hydroxyanthranilic acid (3-HAA) and anthranilic acid (AA) were determined by HPLC and quantified using a sequential diode-array UV and fluorescence detection as previously described (Guillemin et al., 2007). The HPLC analysis was carried out in an uHPLC system (Agilent 1290 Infinity, CA, United States) by using an Agilent ZORBAX Rapid Resolution High Definition C18, reversed phase column (2.1 × 150 mm, 1.8 μm, Agilent Technologies, CA, United States). The temperature of the column compartment was set at 38°C. Injection volume was 20 μL and the autosampler tray temperature was set at 4^*o*^C to prevent sample degradation. The flow rate was set at 0.75 mL/min with an isocratic elution of 100% of 100 mM sodium acetate, pH 4.65. The identification and quantification of XA, KYN and 3-HK were performed by a UV detector (G4212A, Agilent, CA, United States) with absorbance at 250 nm and a reference signal at 350 nm for XA; and with absorbance at 365 nm and reference signal ‘off’ for KYN and 3-HK. The identification and quantification of TRP, 3-HAA and AA were performed by a fluorescence detector (G1321B xenon flash lamp, Agilent, CA, United States) with an emission wavelength of 438 nm and an excitation wavelength of 280 nm for TRP and 320 nm for 3-HAA and AA. The results were calculated by interpolation using a 6-point calibration curve and expressed as μmol/L or nmol/L.

NEO concentrations in serum samples were determined as previously described with some modifications (de Paula Martins et al., 2018). The same HPLC system and column described previously were used with a mobile phase of 100% of 15 mM potassium phosphate buffer, pH 6.4. The flow rate was set at 0.7 mL/min with an isocratic elution. The identification of NEO was performed by a fluorescence detector (G1321B xenon flash lamp, Agilent, CA, United States) with an emission wavelength of 438 nm and an excitation wavelength of 355 nm. The results were calculated by interpolation using 6-point calibration curve. Levels of NEO were calculated and expressed as nmol/L.

Kynurenic acid (KYNA) concentrations in serum samples were determined by HPLC (Agilent 1260 Infinity, Agilent, CA, United States) and an Agilent ZORBAX Rapid Resolution High Definition C18, reversed phase (4.6 × 100 mm, 3.5 μm, Agilent Technologies, CA, United States). Mobile phase consisted of 95% of 50 mM sodium acetate and 50 mM zinc acetate, pH 5.2 and 5% HPLC grade acetonitrile. The flow rate was set at 1.00 mL/min with an isocratic elution. The identification of KYNA was performed by a fluorescence detector (G1321B xenon flash lamp, Agilent, CA, United States) with emission wavelength of 388 nm and an excitation wavelength of 344 nm. The results were calculated by interpolation using a 6-point calibration curve. Levels of KYNA were calculated and expressed as nmol/L.

### Metabolites Quantification by Gas Chromatography/Mass Spectrometry

QUIN and picolinic acid (PIC) concentrations in serum samples were determined using an Agilent 7890 gas chromatograph coupled with an Agilent 5975 mass spectrometer following a protocol previously described (Guillemin et al., 2007). Briefly, deproteinised-serum samples and deuterated internal standards were dried under vacuum and derivatized with trifluoroacetic anhydride and 1,1,1,3,3,3-hexafluoroisopropanol for an hour at 60 ^*o*^C. Fluorinated esters were then extracted into toluene and washed with 5% sodium bicarbonate. The upper organic layer was collected and washed with 1 mL MilliQ water, and dried using sodium sulfate packed pipette tips. Samples were then injected under a splitless mode onto a HP-5MS GC capillary column (Agilent, CA, United States) and the analysis was carried out with the MS operating in negative chemical ionization mode. Selected ions (m/z 273 for PIC, m/z 277 for 4-PIC, m/z 467 for QUIN and m/z 470 for d3-QUIN) were simultaneously monitored. GC oven settings were as follows: oven temperature was held at 75°C for 3 min and then ramped to 290°C at a rate of 25°C/min and held at 290°C for 4 min for a total run time of 15.6 min. Quantification was achieved through normalization with respect to the internal standards and interpolation using 6-point calibration curves for each metabolite. Levels were calculated and expressed as nmol/L.

### BH4 Quantification by ELISA

BH4 concentrations in serum samples were assessed by ELISA, using a commercial kit (Novus Biologicals, Colorado, United States), and following the manufacturer’s instructions. The levels of BH4 were estimated by interpolation from a standard curve by colorimetric measurements at 450 nm on a plate reader (PHERAstar^®^ *FSX*, Offenburg, Germany). Results were calculated as nmol of BH4 per liter (nmol/L).

### Cytokine Analysis

Aliquoted serum samples were thawed and filtered 0.22μm before being analyzed in duplicate by Eve Technologies (Calgary, AB, Canada) using a Milliplex human high sensitivity T-cell discovery array 14-plex assay kit (Millipore). Sample concentrations of the 14 analytes were determined using a 7-point standard curve using the manufacturer’s software.

### Statistical Analysis

Fisher’s exact test was used to compare the proportion of male and female participants between the groups (Prism 6, GraphPad Software Inc.). All other data were first analyzed for the presence of multiple outliers using ROUT test and all the outliers were excluded. Subsequently, the data were analyzed for normality using the D’Agostino-Pearson normality test. Unpaired Student’s *T*-test (for normally distributed data), or a Mann–Whitney *U*-test (for non-normally distributed data) were used to test for statistically significant differences between the groups. Unpaired and two-tailed tests were used, and *P* < 0.05 was considered significant for these group comparisons. The relationship of pain and psychological variables, and cytokine levels to KYN- and BH4- metabolites was assessed using linear regression analyses, with the family-wise error rate corrected using the Benjamini-Hochberg procedure, at a false discovery rate of *q* < 0.1. This is the most appropriate and stringent methodology to correct for multiple comparisons and has been used by similar studies (Luchting et al., 2015; Russo et al., 2019). Only relationships that were significant following this correction are reported.

## Results

The demographics, clinical and psychological measures in DNP and DC participants are shown in Table 1. Among patients who had type 1 diabetes mellitus, 61.9% (*n* = 13) had DN4 scores ≥ 4 and presented with persistent pain for at least 3 months, meeting the criteria for chronic DNP (Bouhassira et al., 2005). This matched the clinical diagnosis of diabetic painful peripheral neuropathy in this group. The average time since pain onset in the DNP group was 9.08 years. All 13 DNP patients had pain in both lower limbs, 3 had pain in the left upper limb, and 2 had pain in the right upper limb. All of the DC group reported no neuropathic pain symptoms, scoring 0 on DN4, nor did they have any sensory deficits in the limbs or have any microvascular complications of diabetes. Therefore, we confirmed that there was no evidence of peripheral neuropathy (painful or otherwise) in the diabetic control group, but clear evidence of painful peripheral neuropathy in the DNP group.

**TABLE 1:** Demographics, clinical and psychological measures in type 1 diabetic neuropathic pain and type 1 diabetic control groups.

		**DC(*n* = 8)**	**DNP(*n* = 13)**
Sex (F/M)		4/4	5/8
Age (years)		54.1 (43 – 70)	60.4 (40 – 71)
Painful peripheral neuropathy onset (years ago)		n/a	9.08 ± 9.6
Lower limbs affected (L/R)		n/a	13/13
Upper limbs affected (L/R)		n/a	3/2
DN4 Score (0–10)		0	5.69 ± 1.44
BMI (kg m^–2^)		30 (29 – 37)	34.9 (25.5 – 50.1)
Pain score (VAS 0–100)		3.8 ± 3.7	55.8 ± 24.4***
SF-MPQ-2 (0–220)		1.5 ± 2	95.9 ± 45.8**
DASS 21 (0–42)	Depression	2.5 ± 3.3	13.2 ± 10.6**
	Anxiety	2.2 ± 3.7	12 ± 8.7**
	Stress	7.2 ± 9.2	14.6 ± 10.2
TSK (0–68)		29.6 ± 3.8	44.3 ± 6.3***
PCS (0–52)		1.8 ± 2.8	29.4 ± 15.1***
PSEQ (60–0)		57.2 ± 4.4	39.5 ± 13.7***

DN4, Douleur Neuropathique 4 questionnaire; VAS, Visual analog scale; SF-MPQ-2, Short-form McGill Pain Questionnaire; DASS21, Short-form Depression, Anxiety and Stress Scale; TSK, Tampa Scale for Kinesiophobia; PCS, Pain Catastrophizing Scale; PSEQ, Pain Self-Efficacy Questionnaire. Data are presented as mean ± standard deviation (X ± SD). **P < 0.01, ***P < 0.001, unpaired two-tailed Mann–Whitney *U*-test.

The proportion of females in the DNP group was greater than in the DC group, but this was not statistically significant (*P* = 0.67). Severe pain was confirmed in the DNP participants using the VAS pain scale (*U* = 0, *P* < 0.001) and the SF-MPQ-2 questionnaire (*U* = 0, *P* < 0.001). Psychological profiles were examined using the DASS21 scale, which showed moderate depression (*U* = 14, *P* < 0.01), moderate anxiety (*U* = 15, *P* < 0.01), and mild stress (*U* = 26, *P* = 0.06) in the DNP group. Kinesiophobia, pain catastrophizing and reduced pain self-efficacy were also found in the DNP group, which had significantly higher scores on the TSK (*U* = 3, *P* < 0.001), and PCS (*U* = 1.5, *P* < 0.001) scales, and lower scores in the PSEQ scale (*U* = 6, *P* < 0.001).

**FIGURE 2:**
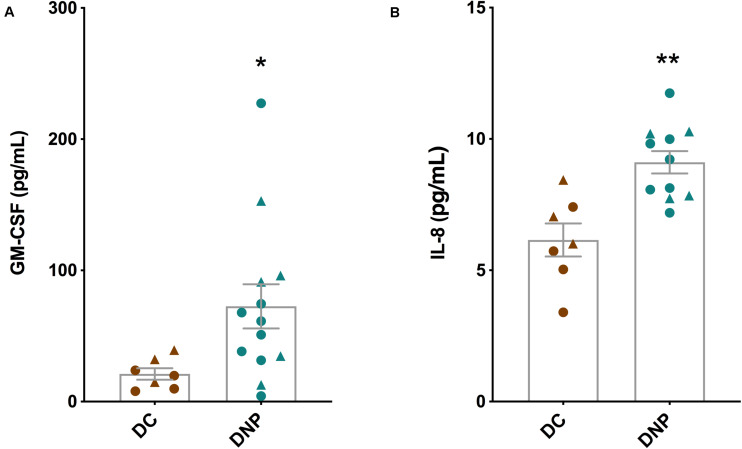
Cytokine levels in the serum of diabetic neuropathic pain and diabetic control groups. **(A)** GM-CSF and **(B)** IL-8 in serum of DNP participants and diabetic controls. *P < 0.05, **P < 0.01, unpaired two-tailed Mann–Whitney *U*-test. Circles represent male participants; triangles represent female participants.

The serum protein levels of a panel of 14 cytokines were quantified in the DNP and DC groups (Table 2). Of particular interest are the significant increases in the levels of inflammatory cytokines GM-CSF (*U* = 16, *P* < 0.05, Figure 2A) and IL-8 (*U* = 6, *P* < 0.01, Figure 2B) in the DNP group compared to controls. There was no obvious pattern of differential cytokine levels between male and female participants. None of the other 12 cytokines showed significant differences between the two groups, although it is important to highlight that major pro-inflammatory cytokines, such as IFN-γ, TNF-α, IL-1b, IL-2, IL-17A, and IL-23 were highest in the DNP, whereas anti-inflammatory cytokines, IL-4 and IL-10, were lowest in the DNP group. The significant increases in IL-8 and GM-CSF suggest the presence of an inflammatory state in the DNP group.

**TABLE 2:** Cytokine protein levels in the serum of diabetic neuropathic pain and diabetic control groups.

**Cytokine(pg/mL)**	**DC (X ± SD; *n* = 8)**	**DNP (X ± SD; *n* = 13)**
GM-CSF	21.1 ± 11.6	72.6 ± 60.9*
IFN-γ	10.6 ± 6.8	14.6 ± 10
TNF-α	7.4 ± 3.4	8.1 ± 4.8
IL-1b	1.6 ± 0.6	2.5 ± 1.5
IL-2	3.8 ± 1.9	7.1 ± 4.4
IL-4	7.3 ± 7.7	6.6 ± 4.3
IL-5	1.5 ± 0.9	1.7 ± 0.7
IL-6	1.6 ± 0.9	1.8 ± 0.7
IL-8	6.1 ± 1.6	9.1 ± 1.4**
IL-10	5.4 ± 3.0	4.9 ± 3.3
IL-12	3.4 ± 1.8	4.1 ± 2.4
IL-13	4.9 ± 2.9	3.8 ± 1.9
IL-17A	13.5 ± 8.1	18.3 ± 11.6
IL-23	323.4 ± 230.9	474.2 ± 358.0

*P < 0.05, **P < 0.01, unpaired two-tailed Mann–Whitney *U*-test.

**FIGURE 3:**
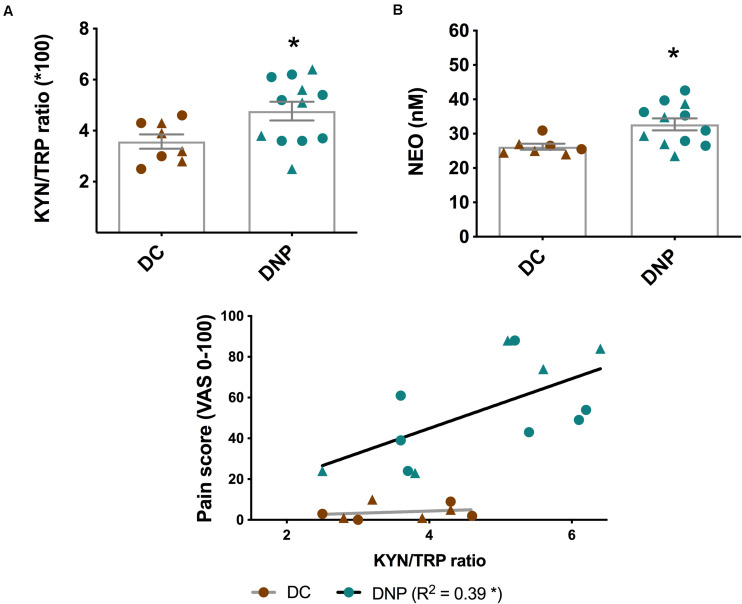
Levels of two major inflammatory biomarkers in the serum of diabetic neuropathic pain and diabetes control groups. **(A)** KYN/TRP ratio and **(B)** NEO in serum of DNP participants and diabetic controls. *P < 0.05, unpaired two-tailed Student’s *T*-test. **(C)** The relationship between pain intensity score and the KYN/TRP ratio in DNP and DC groups. *P < 0.05 & R^2^ linear regression analysis. Circles represent male participants; triangles represent female participants. TRP, Tryptophan; KYN, Kynurenine; NEO, neopterin; VAS, Visual analog scale.

This is further supported by a significant increase in the KYN/TRP ratio in the DNP group [*t*_(__18__)_ = 2.348, *P* < 0.05] (Figure 3A). The KYN/TRP ratio is an indirect marker of the enzymatic activity of IDO1, which is activated by pro-inflammatory cytokines. Furthermore, the KYN/TRP ratio positively correlated with pain intensity in the DNP group (*P* < 0.05, *R^2^* = 0.39), but not in diabetic controls (Figure 3C). There was no clear pattern of differential KYN/TRP ratio between male and female participants. Overall, these findings provide support that there is increased inflammatory activation in individuals with diabetic neuropathic pain.

Metabolites of the KP and BH4 pathway have been quantified in the serum of the DNP and diabetic control participants (Table 3). No significant alterations were observed in the different metabolic intermediates or end products of the KP. Lower levels of TRP and higher levels of KYN in the DNP group support the aforementioned significant increase in the KYN/TRP ratio (Figure 3A). The neurotoxic metabolite QUIN was 248.8 ± 191.2 nmol/L in the DNP group and 187.6 ± 110.6 nmol/L in the DC group, however this difference failed to reach significance due to high levels of individual variation [*t*_(__19__)_ = 1.303, *P* = 0.21]. While BH4 levels were unaltered, the levels of the macrophage produced inflammatory biomarker NEO were significantly increased in the serum of DNP compared to DC participants [*t*_(__17__)_ = 2.717, *P* < 0.05] (Figure 3B and Table 3).

**TABLE 3:** Levels of KYN and BH4 pathway metabolites in diabetic neuropathic pain and diabetic control groups.

**Metabolite**	**DC** (**X ± SD; *n* = 8)**	**DNP (X ± SD; *n* = 13)**
TRP (μmol/L)	52.96.1	46.87.1
KYN (μmol/L)	1.80.4	2.20.5
3-HAA (nmol/L)	18.83.6	19.46
AA (nmol/L)	15.13.5	15.053.9
KYNA (nmol/L)	30.410.4	27.314.7
3-HK (nmol/L)	14.54.5	12.13.5
XA (nmol/L)	9.31.7	11.12.5
PIC (nmol/L)	29.015.4	31.912.6
QUIN (nmol/L)	187.6110.6	248.8191.2
BH4 (nmol/L)	2.5750.6076	2.4580.4672
NEO (nmol/L)	26.200.8794	32.721.741*

TRP, Tryptophan; KYN, Kynurenine; 3-HAA, 3-Hydroxyanthranilic acid; AA, Anthranilic acid (AA); KYNA, Kynurenine acid; 3-HK, 3-Hydroxykynurenine; XA, Xanthurenic acid; QUIN, Quinolinic acid; PIC, Picolinic acid; BH4, Tetrahydrobiopterin; NEO, Neopterin. All data are presented as mean ± standard deviation (X ± SD). *P < 0.05, unpaired two-tailed Student’s *T*-test.

In addition to the elevated activity of IDO1 predicted by the ratio of TRP/KYN, the activity of the other KP enzymes were estimated by the product/substrate ratios (Table 4). The kynurenine aminotransferase (KAT) activity responsible for the production of XA from the substrate 3-HK (identified here as KAT B) was significantly higher (*U* = 9, *P* < 0.01) in the DNP group. This is in line with lower levels of 3-HK and higher levels of XA (Table 3). Furthermore, the total KAT activity (calculated as the sum of KAT A and KAT B activities) was also significantly higher (*U* = 15, *P* < 0.05) in DNP participants, however given KAT A activity is not significantly different this is most likely the result of a significant change in KAT B. No other alterations were observed in the activity of the different enzymes of the KP.

**TABLE 4:** Activity of KP enzymes in the serum of DNP and diabetic control participants.

**Enzymes**	**Expression**	**DC (X ± SD; *n* = 8)**	**DNP (X ± SD; *n* = 13)**
KMO	100 × 3-HK/KYN	0.80.3	0.580.2
KAT A	100 × KYNA/KYN	1.60.4	1.20.4
KAT B	100 × XA/3-HK	56.214	11150**
Total KAT	KAT A + KAT B	57.814	112.250**
KYNU A	100 × AA/KYN	0.810.1	0.730.2
KYNU B	100 × 3-HAA/3-HK	148.773	166.271
Total KYNU	KYNU A + KYNU B	149.574	167.071

KMO, kynurenine 3-monooxygenase; KYNU, kynureninase; KAT, kynurenine aminotransferases; KYN, Kynurenine; 3-HAA, 3-Hydroxyanthranilic acid; AA, Anthranilic acid (AA); KYNA, Kynurenine acid; 3-HK, 3-Hydroxykynurenine; XA, Xanthurenic acid. All data are presented as mean ± standard deviation (X ± SD). *P < 0.05, **P < 0.01, unpaired two-tailed Mann–Whitney *U*-test.

**FIGURE 4:**
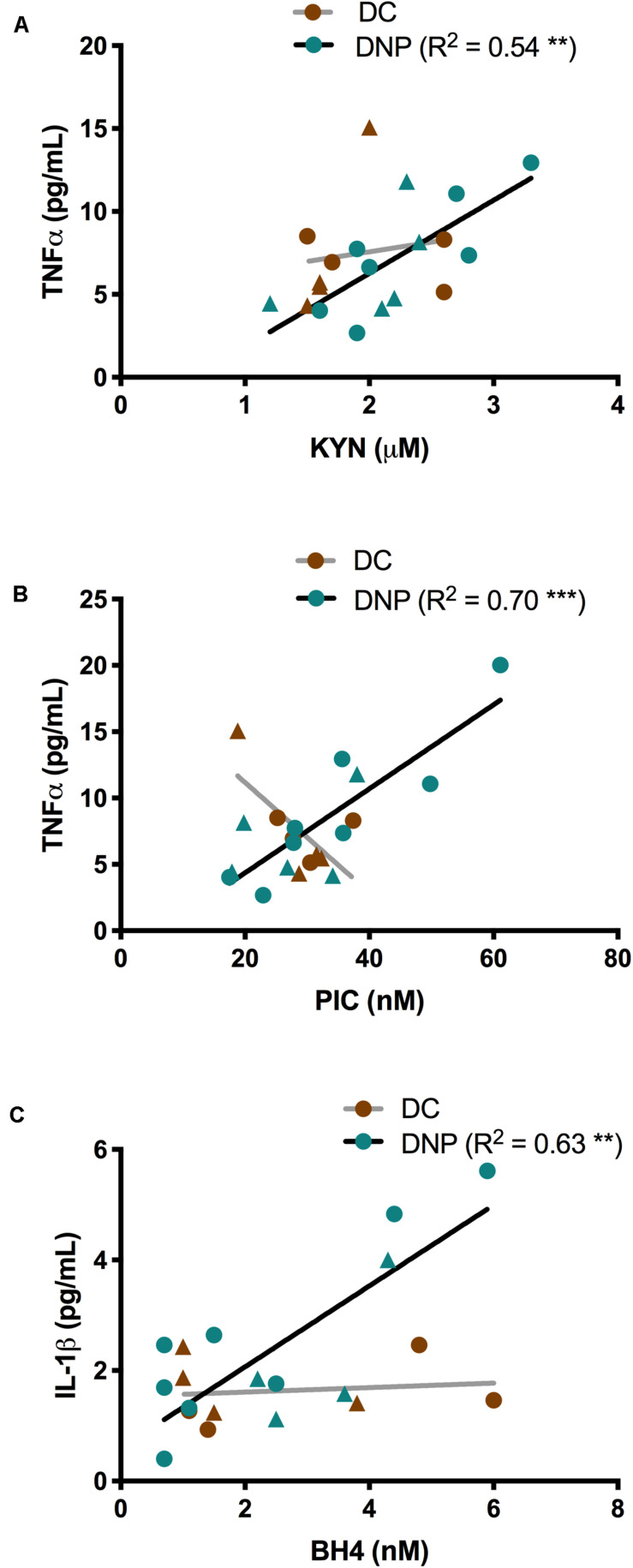
The relationships between TNF-α and IL-1β, and metabolites of the KYN and BH4 pathways in serum of diabetic neuropathic pain and diabetic control groups. **(A)** TNF-α and KYN expression correlate in the DNP group. **(B)** TNF-α and PIC expression correlate in the DNP group. **(C)** IL-1β and BH4 levels correlate in the DNP group. **P < 0.01, *P* < 0.001 & R^2^ from linear regression analysis. Circles represent male participants; triangles represent female participants.

Given the known relationship between inflammatory cytokines and the KP and BH4 metabolites (Figure 1), linear regression was performed separately within the DNP and DC groups to uncover any relationships. Significant relationships were identified between inflammatory cytokines TNF-α and IL-1β and metabolites KYN, PIC and BH4 in the DNP group, but not the DC group (Figure 4). There were significant positive correlations between KYN (*P* < 0.01, *R^2^* = 0.54, Figure 4A) and PIC (*P* < 0.001, *R^2^* = 0.70, Figure 4B) with TNF-α within the DNP group. There was a significant positive correlation between IL-1β and BH4 in the DNP group (*P* < 0.01, *R^2^* = 0.63, Figure 4C). These relationships exist in the DNP group despite the fact that none of these markers were significantly elevated compared to DC (Tables 2, 3). There were no obvious patterns between males and females. Overall, these findings suggest some degree of individual variation across the DNP group and that increased pro-inflammatory cytokine levels could be responsible for activation of the KP and BH4 pathway in some DNP participants.

## Discussion

In this study, we have demonstrated that the pro-inflammatory cytokines GM-CSF and IL-8, and the inflammatory biomarkers NEO and the KYN/TRP ratio, were elevated in participants with type 1 diabetes mellitus and neuropathic pain. The serum levels of a number of other pro-inflammatory cytokines positively correlated with metabolites of the KYN- and BH4- pathways, suggesting that immune mediators drive activation of these pathways.

It has been well documented that the appearance of peripheral neuropathy in type 1 and type 2 diabetes mellitus is strongly associated with increases in inflammatory biomarkers, including C-reactive protein, TNF-α, and IL-6 (Hussain et al., 2013; Zhu et al., 2015; Ge et al., 2016). Here, we specifically examined the serum levels of cytokines in participants with type 1 diabetes mellitus complicated by neuropathic pain (DNP group). We found that GM-CSF and IL-8 were elevated in the DNP group. GM-CSF promotes the survival and activation of macrophages, neutrophils and eosinophils, and has been shown to polarize macrophages toward an M1 pro-inflammatory phenotype (Hamilton, 2008). It has also been shown to sensitize peripheral nociceptors indirectly through the activation of macrophages, and cause sprouting of sensory nerve endings in the skin (Schweizerhof et al., 2009; Tewari et al., 2020). A recent study has confirmed a role for GM-CSF in neuropathic pain, where it can potentiate the release of pro-inflammatory mediators from spinal glial cells (Nicol et al., 2018). Moreover, GM-CSF was identified as an important biomarker in our recent study in complex regional pain syndrome (Russo et al., 2020). The chemokine IL-8 is predominantly released from macrophages, recruits both neutrophils and T lymphocytes, and has recently been associated with neuropathic pain in burning mouth syndrome and peripheral neuropathies (Barry et al., 2018; Langjahr et al., 2018). An imbalance of pro- and anti-inflammatory cytokines has previously been reported in other painful peripheral neuropathies (Uceyler et al., 2007). The mean serum level of IFN-γ was ∼40% higher in the DNP group compared to the control group (see Table 2), and although not a significant increase, it could be biological important as it is a major activator of IDO1. The cytokine profiles of DNP participants suggest an inflammatory state, involving GM-CSF and IL-8, may play a role in the development of neuropathic pain in type 1 diabetes mellitus. Given these cytokines may act indirectly through the activation of immune cells, particularly macrophages, an immunophenotyping study of blood from diabetics with neuropathic pain would shed further light on these findings.

Under inflammatory conditions, the rate-limiting enzyme of BH4 biosynthesis, GTP cyclohydrolase I (GTPCH), is upregulated up to 100-fold (Shi et al., 2004). This is in keeping with our observation of a strong positive relationship between BH4 serum levels and the pro-inflammatory cytokine IL-1β in the DNP group. This suggests that BH4 may be increased in individuals with higher levels of IL-1β despite not being significantly elevated in all DNP participants (Werner et al., 1990; Shi et al., 2004). Several pre-clinical studies have identified BH4 as a key mediator of chronic pain (Latremoliere et al., 2015; Fujita et al., 2019). The activity of PTPS and sepiapterin reductase, enzymes of the BH4 pathway downstream of GTPCH, are only slightly increased by inflammation, with the metabolic blockage resulting in the accumulation of NEO. NEO is therefore considered a far more sensitive biomarker for inflammatory activation, and proxy for GTPCH activity, than BH4 (Werner et al., 1990, 1993; Ghisoni et al., 2015). NEO is a macrophage activation marker, produced by macrophages stimulated by the Th1 T lymphocyte derived cytokine, IFN-γ (Huber et al., 1983), and is elevated in the dorsal root ganglia (DRG) (Tegeder et al., 2006). A study in HIV-infected patients with peripheral neuropathy found an increase in blood and CSF NEO levels, compared to HIV patients without neuropathy, together with an increase in CD14 + CD16 + monocytes (Wang et al., 2014). Given the significant increases in macrophage-related cytokines, GM-CSF and IL-8, in the DNP group, and the link to nerve injury and neuropathy, it is perhaps unsurprising that we were also able to observe an increase in NEO levels in the DNP group. These findings make NEO a potentially useful biomarker in diabetic neuropathic pain.

Two major enzymes catabolize TRP via the KP: tryptophan (2,3)-dioxygenase (TDO) in the liver, and IDO1 elsewhere (Schrocksnadel et al., 2006). The extrahepatic KP enzyme, IDO1, contributes very little (< 2%) to TRP degradation under normal conditions, however it assumes a greater quantitative significance after activation by cytokines, such as IFN-γ and TNF-α, and results in a decrease of TRP and an increase of KYN concentration in blood (Schrocksnadel et al., 2006). As a result, the KYN/TRP ratio is a well-established marker for IDO1 and inflammatory activation, with an elevated ratio observed in both chronic lower back pain and complex regional pain syndrome (Kim et al., 2012; Alexander et al., 2013). Here, we observed an increase in the KYN/TRP ratio in the DNP group compared to pain-free diabetic controls. Given the positive relationship between the KYN/TRP ratio and the pain intensity score, the KP is a candidate for direct involvement in pathological processes leading to diabetic neuropathic pain. However, we caution that an important follow up study should be undertaken to directly confirm an increase in IDO1 activity and mRNA levels in peripheral blood mononuclear cells from diabetics with neuropathic pain. Interestingly, KYN and PIC showed a significant positive correlation with the serum level of TNF-α in DNP, suggesting that TNF-α levels may be responsible for individual differences in KYN and PIC levels in the DNP group. A previous study showed that TNF-α works synergistically with IFN-γ to induce IDO1 expression, which increases production of KYN and other downstream KP metabolites (Pemberton et al., 1997; Robinson et al., 2003). Little is known about the physiological role of PIC, however a neuroprotective role has been suggested (Cockhill et al., 1992; Beninger et al., 1994). In summary, levels of GM-CSF and IL-8 were higher in the DNP group, and TNF-α and IL-1β positively correlated with KYN, PIC and BH4 levels, supporting the idea that immune activation leads to pathological activation of the KYN and BH4 pathways.

The pivotal metabolite KYN can be metabolized by different enzymes along distinct routes to produce several neuroactive metabolites that have been implicated in immune regulation and tolerance mechanisms (Munn et al., 1998). Evidence from pre-clinical studies has revealed the contribution of KP enzymes within the DRG, the spinal cord and the hippocampus in the development of neuropathic pain (Zhou et al., 2015; Rojewska et al., 2016, 2018; Laumet et al., 2017). An up-regulation of IDO1 and KMO in DRG and spinal cord neurons was associated with allodynia and hypersensitivity in a neuropathic pain model (Rojewska et al., 2016, 2018). Concurrently, the administration of KMO inhibitors (Ro61-6048 and JM6) or IDO1 inhibitor (1-methyl-D-tryptophan) decreased the pain hypersensitivity (Rojewska et al., 2016, 2018).

The present study suggests an activation of the KP with a possible conversion into QUIN and XA production in participants with type 1 diabetes mellitus and neuropathic pain. The QUIN serum levels were ∼30% higher in the DNP group than in the diabetic control group, and although this difference was not significant due to high variance amongst individuals, it provides the impetus for a larger follow up study. Similarly, our data demonstrated XA serum concentration was ∼20% higher in diabetes participants with neuropathic pain than in the diabetes pain-free control group, and this observation is supported by a significant increase in KAT activity, the enzyme responsible for the XA production. Furthermore, QUIN and XA were commonly elevated biomarkers in samples from a large cohort of patients with chronic pain (Gunn et al., 2020). Given these KP metabolites (i.e., QUIN and XA) are neuroactive compounds that act via glutamatergic receptors (Guillemin, 2012; Neale et al., 2013), further studies exploring their relevance for inflammation-induced pain hypersensitivity would shed further light on these findings.

### Study Limitations

The limitations of this study are the small cohorts, and despite being non-significant, the different proportion of females and males in each group. In particular, sex differences in immune function and pain processing could represent a confounding factor in our findings (see review Mapplebeck et al., 2016), especially given sex differences in TRP and KYN metabolite serum levels (Badawy and Dougherty, 2016). The most significant limitation of our study is that subjects were not fasted prior to obtaining blood samples, which could lead to fluctuations in levels of some metabolites through food intake and dietary components (Badawy, 2010). Also, future studies should control for TRP and KYN modulators such as glucocorticoids and insulin, which play an important role in lowering serum TRP levels (Badawy, 2017). It is possible that the 7-day drug wash-out could leave residual effects on the immune system, since although myeloid cells have a rapid turnover, lymphocyte turnover is much slower, only 2% daily (De Boer et al., 2003).

## Conclusion

In summary, our results suggest that inflammatory activation through elevated pro-inflammatory cytokines, neopterin and upregulation of the kynurenine pathway might be involved in the pathophysiology that leads to the appearance of neuropathic pain in type 1 diabetes mellitus. Our research encourages further studies exploring the role of neuro-immune interactions, particularly through the cytokines GM-CSF and IL-8 as well as the kynurenine and tetrahydrobiopterin pathways, in chronic pain conditions.

## Data Availability Statement

All raw datasets generated for this study are available on reasonable request from the corresponding author.

## Ethics Statement

The studies involving human participants were reviewed and approved by Human Ethics Committee from University of Sydney (HREC #2017/019) and Macquarie University (HREC #5201600401). The patients/participants provided their written informed consent to participate in this study.

## Author Contributions

AS: acquisition of data, data curation, and writing the original draft. BH: acquisition of data, data curation, and revising the article. VT: investigator of the kynurenine and tetrahydrobiopterin analysis and revising the article. AL: intellectual and technical support for tetrahydrobiopterin pathway analysis and revising the article. MR: conception of the study, participant recruitment, and funding acquisition. DS: participant recruitment, clinical database management, and revising the article. DB: participant recruitment, sample collection, and clinical database management. KW: participant recruitment and revising the article. JO’B: sample preparation and revising the article. GG: principle investigator of the kynurenine and tetrahydrobiopterin analysis, funding acquisition, and revising the article. PA: conception of the study, project supervisor, funding acquisition, data curation, writing the original draft, and revising the article. All authors contributed to the article and approved the submitted version.

## Conflict of Interest

The authors declare that the research was conducted in the absence of any commercial or financial relationships that could be construed as a potential conflict of interest.
